# *Rhizobium* biostimulation of blackberry modulates survival pathways in *Caenorhabditis elegans* across biological kingdoms

**DOI:** 10.1038/s41538-025-00525-5

**Published:** 2025-07-29

**Authors:** Rocío Roca-Couso, José David Flores-Félix, Begoña Ayuda-Durán, Rebeca Ferreras-Charro, Ignacio García-Estévez, Paula García-Fraile, Raúl Rivas

**Affiliations:** 1 Department of Microbiology and Genetics, Edificio Departamental de Biología, Universidad de Salamanca, Salamanca, Spain; 2 Institute for Agribiotechnology Research (CIALE), Salamanca, Spain; 3 Department of Analytical Chemistry, Nutrition and Food Science, Universidad de Salamanca, Salamanca, Spain; 4 Associated Unit, University of Salamanca-CSIC (IRNASA), Salamanca, Spain

**Keywords:** Applied microbiology, Quality of life, Ageing

## Abstract

Endophytic *Rhizobium* species represent promising bioinoculants for enhancing crop performance and nutritional profiles. This study investigated the impact of *Rhizobium* sp. CRRU65 inoculation on blackberry (*Rubus* sp.) plants, with emphasis on fruit phytochemical composition and cross-kingdom bioactivity. Inoculated plants exhibited a significant increase in yield and elevated levels of phenolic compounds, notably sanguiin H6 and cyanidin-3-O-glucoside, as quantified by HPLC-DAD-MS. Antioxidant functionality was evaluated using *Caenorhabditis elegans* under oxidative stress. Extracts from inoculated fruits significantly improved nematode survival, accompanied by transcriptional upregulation of *skn-1* and *hsp-16*, genes involved in stress response and proteostasis. These findings demonstrate that *Rhizobium* sp. CRRU65 enhances not only agronomic traits but also the nutraceutical quality of blackberry fruits, with beneficial effects extending across biological kingdoms. This work underscores the potential of endophytic bacteria to contribute to sustainable agriculture and functional food innovation through molecular and physiological modulation in both plants and animal models.

## Introduction

Endophytic bacteria have emerged as a novel and sustainable approach to enhancing crop efficiency, avoiding many of the drawbacks associated with conventional methods^1^. These bacteria are known for their Plant Growth Promotion (PGP) mechanisms, which play a crucial role in improving plant development and fitness. Among them, those belonging to the genus *Rhizobium* are particularly remarkable^2^. Bacteria belonging to this genus have been extensively studied demonstrating their effect in improving crop production since their application produces an increment on flowering and/or fruiting^3–5^. Beyond the increment in crop’s productivity, recent studies suggest that their inoculation can alter fruits’ composition, potentially enhancing nutraceutical value^6^. Thus, *Rhizobium* spp. inoculation of tomato, lettuce or strawberry crops showed an increment in carotenes and phenolic compounds^2,4,7^. In this sense, this approach may be of special interest for functional foods. These foods are rich in bioactive compounds such as dietary fiber, phenolic compounds and vitamins that make them a source of health benefits beyond basic nutritional requirements^8^. Within this framework, blackberries (*Rubus ulmifolius* Schott) garnered attention due to their significant health benefits related to their profile of bioactive compounds, including flavonoids, such as anthocyanins, and dietary fiber, driving the expansion of their industrial production^9^. The composition and concentration of these compounds can vary depending on several factors such as plant species, location, cultivar, or harvest time^10^. However, the most abundant phenolic compounds in blackberries are phenolic acids, flavonoids and tannins^11^. This phenolic profile is considered responsible for the beneficial effects associated with blackberry consumption, such as anticancer, antibacterial and anti-inflammatory activities^12^.

Studies based on the bioactive properties of fruits have been conducted using model organisms such as *Caenorhabditis elegans*^10,13^. This organism is used for developmental studies, neurobiology, and evaluating pharmacological and toxicological effects in humans since it shares approximately 60% to 80% of their genes with humans and many basic physiological processes and oxidative stress responses of higher organisms are highly conserved^14,15^. Additionally, this organism is also used for studying bioactive effects of phytochemicals of foods and plants^13^.

Considering previous studies reporting that *Rhizobium* inoculation increases bioactive compounds in certain crops such as lettuce, mango or blueberry^2,3,6^, this work focuses on the use of an endophytic *Rhizobium* strain isolated from blackberry roots and its effect on blackberry crops. Fruit production and phenolic content are evaluated after inoculation. Additionally, given the observed increment in bioactive compounds, this work evaluates the potential impact of these compounds on the survival rate under oxidative stress by using *C. elegans* as a model organism. This approach provides continuity by linking the enhancement of bioactive compounds to their potential health-related effects, as suggested in the context of the previous studies.

## Results

### Bacteria identification

Species identification by 16S rRNA gene sequencing showed that CRRU65 strain had a similarity of 100% with respect to *Rhizobium laguerreae* FB206^T^. For species differentiation, dDDH between our strain and the ten most closed species were calculated (Table S1). Results showed that, in all cases, dDDH values were below 70%, indicating CRRU65 belonged to an undescribed species. That is why, CRRU65 strain was identified as *Rhizobium* sp. CRRU65.

### PGP evaluation and strain selection

In vitro evaluation of PGP mechanisms provides an overall impression of the isolate’s potential to improve plant development. Evaluation of several PGP traits showed a solubilization halo in both Pikovskaya agar medium supplemented with Ca_2_PO_3_ and Aleksandrov medium. However, there were no halos in the rest of the phosphate solubilization media nor the siderophore medium. Regarding phytohormones production, *Rhizobium* sp. CRRU65 produced 62.0 mg L^−1^ of indole-like compounds, when grown in JMM liquid medium supplemented with tryptophan, whereas when measuring the concentration of this phytohormone by HPLC, the result obtained were 0.22 mg L^−1^. These results suggested that *Rhizobium* sp. CRRU65 could possess a high potential in improving crop yield and quality, although the subsequent verification in plant assays is required.

### Genome analysis

In silico studies of genomes may provide valuable information about bacterial potential. Thus, genomic sequencing became a fundamental tool for the analysis and improvement of knowledge related to microbiological processes, such as microorganism–plant interactions. In this sense*, Rhizobium* sp. CRRU65 genome was sequenced and annotated. The general characteristics of the genome are presented in Table S1.

The analysis of the genome provided additional information to the tests carried out in vitro, elucidating the possible mechanisms that could be involved in the promotion of flowering, fruit ripening, and increasing plant phenolic content.

The genome of *Rhizobium* sp. CRRU65 contains tRNA dimemthallyl transferase (*miaA*), 2-methylthio N6-dimemthallyladenosne synthase (*miaB*), cytokinin monophosphate phosphoribo-hydrolase (*yvdD)*, and diaminopemlate epimerase (*dapF*) genes involved in cytokinin production; *speBC* involved in spermidine and putrescine biosynthesis; and *gsp* genes involved in spermine biosynthesis.

Vitamins are essential compounds for the correct development of plants. Various genes related to the synthesis of these vitamins were annotated in CRRU65 genome such as *thiDEFKLMNO for* vitamin B1 thiamine, *cobACFGLNPQRSTU* for B12 cobalamin, *pdxAHJK* for B6 pyridoxine, *ribABDEFH* for B2 riboflavin, *panBCE* for B5 pantothenate, and *folABCDEKP* for B9 folate.

### Blackberry production assay on greenhouse conditions

Results obtained so far seemed to indicate that *Rhizobium* sp. CRRU65 could influence plant development, specifically flowering, which could involve an increment in fruit production. To test that, we evaluated the effect of the bacteria inoculation on blackberry fruit production under greenhouse conditions. The evaluated parameters were chlorophyll content, number of flowers per plant, number of fruits per plant, average fruit weight per plant, and total fruit weight per plant.

**Fig. 1:**
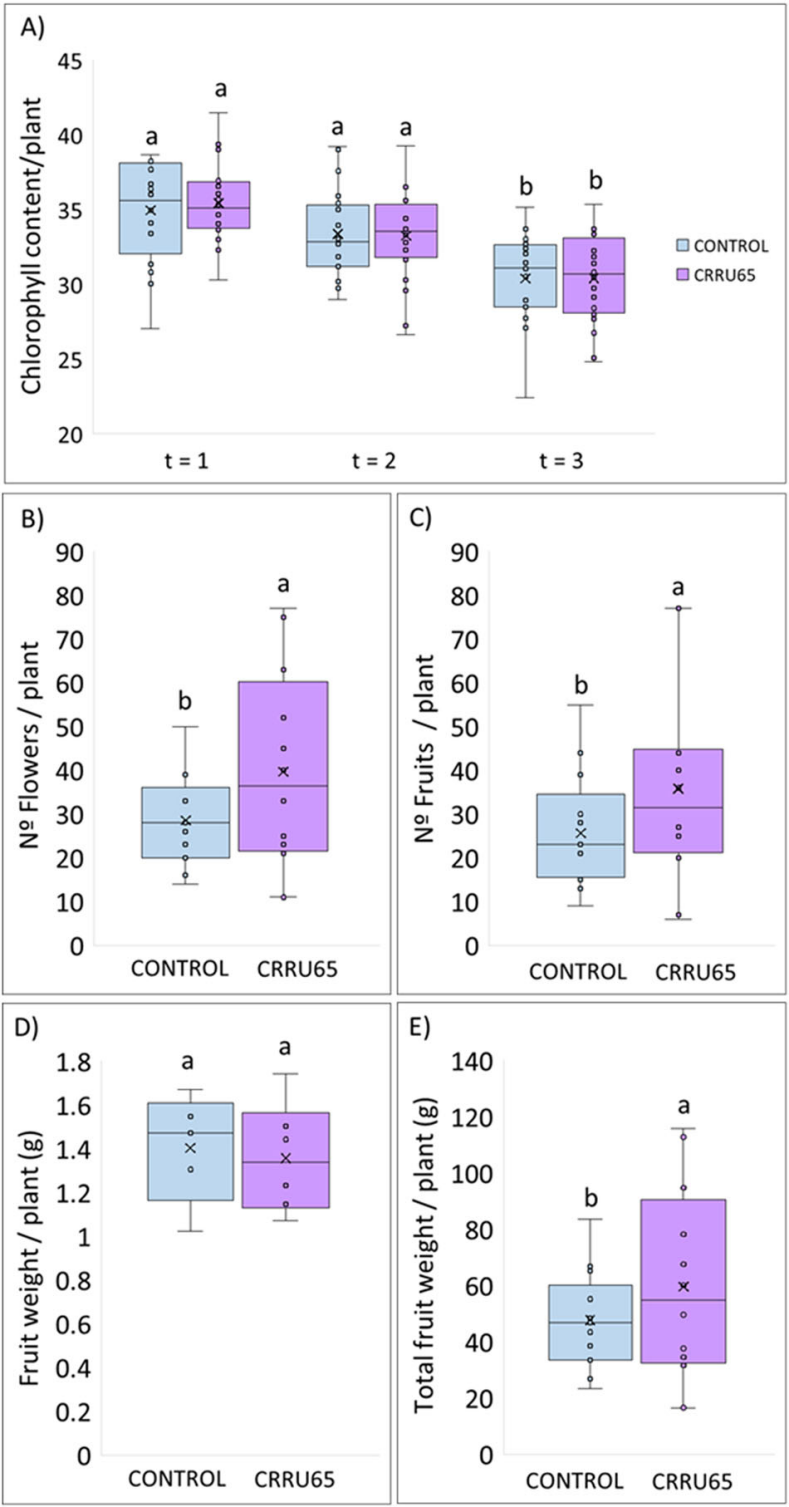
Results of blackberry production on greehouse conditions and vegetative parameters. **A** Average chlorophyll content per treatment per measured time(t = 1: appearance of the first leaf, t = 2: two weeks after t = 1, and t = 3: appearance of the first fruit); **B** Average number of flowers per plant per treatment; **C** Average number of fruits per plant per treatment; **D** Average weight of fruit per treatment; **E** Average weight of the total fruit production per plant. Different letters (a and b) indicate significant differences (*P* ≤ 0.05) between treatments as determined by Tukey test.

The chlorophyll content was measured at three different times: i) after the appearance of the first leaf (t = 1), ii) two weeks after that moment (t = 2) and iii) after the appearance of the first fruit (t = 3). The results showed that the chlorophyll content remained practically constant during the trial, slightly decreasing before fruit production (t = 3) (Fig. 1A).

Regarding flowering, it increased significantly by 128.3% in those plants inoculated with the *Rhizobium* sp. CRRU65 strain (Fig. 1B). Consequently, fruit production significantly increased by 129.1% in those plants inoculated with the *Rhizobium* sp. CRRU65 strain (Fig. 1C–E). Although the average fruit weight remained constant compared to the blackberries collected from the uninoculated control plants, the total fruit yield per plant significantly increased by 124.8%.

### Phenolic composition of blackberries from greenhouse assay

Thus far, *Rhizobium* sp. CRRU65 inoculation of blackberry plants demonstrated an increase in total fruit production, primarily attributed to an increment in the number of flowers. Previous studies reported that *Rhizobium* spp. inoculation enhanced both crop yields and quality^4,6^. In this context, the present study investigated the influence of the inoculation on the phenolic content, as phenolic compounds are among the key bioactive compounds of interest in berry fruits such as blackberry^16^.

To analyze the inoculation effect on fruit quality, once the production reached commercial maturity, the fruits were harvested, and the phenolic composition was analyzed. In the blackberry extracts (BE), we determined one hydroxycinnamic acid, five ellagitannins, one gallotannin, five anthocyanins, one proanthocyanidin, six flavonols, one flavanol, and one flavanone (Table S2). In all samples, the most abundant compound was the ellagitannin sanguiin H6, followed by the anthocyanin cyanidin 3-*O*-glucoside and the hydrolysable tannins trigalloyl HHDP glucoside and glucogallin (Table 1). *Rhizobium* sp. CRRU65 inoculated fruits showed a significant increment of 11.6, 73.7, 19.0, 15.0, and 17.6% in cyanidin 3-*O*-glucoside, kaempferol acetylhexoside, sanguiin H6, sanguiin H6 isomer and trigalloyl HHDP glucoside quantity, respectively. On the contrary, glucogallin quantity showed a decrement of 53.9% in inoculated fruits.

**Table 1:** Phenolic compounds show significant differences in their concentration (µg/g d.w.) between control plants and inoculated plants

Compound	Control	CRRU65	*p* values
Cyanidin 3-*O*-glucoside	41 ± 4 b	46 ± 2 a	0.004
Glucogallin	10 ± 1 a	4.6 ± 0.4 b	0.004
Kaempferol acetylhexoside	0.44 ± 0.08 b	0.77 ± 0.03 a	0.025
Sanguiin H6	181 ± 7 b	215 ± 18 a	0.002
Sanguiin H6 Isomer	384 ± 16 b	442 ± 20 a	>0.001
Trigalloyl HHDP glucoside	20.5 ± 0.5 b	24.3 ± 2 a	0.028

Different letters (a and b) indicate significant differences between treatments as determined by Fisher´s LSD test.

### Thermal stress assay on in vivo model

All of the above-mentioned increased phenolic compounds in blackberries from inoculated plants could contribute to the nutraceutical properties associated with the consumption of this fruit^17,18^. Phenolic compounds contribute to the high antioxidant power of blackberries and some studies suggest that their consumption may reduce the risk of obesity, cardiovascular diseases, degenerative diseases, and various forms of cancer^19^. For instance, the intake of anthocyanins has been associated with a reduction in insulin resistance, a lower risk of myocardial infarction and a moderation of the inflammatory response^20^. In this regard, we conducted an assay using the model organism *C. elegans* to assess whether the differences observed in phenolic compounds concentration between blackberries harvested from plants inoculated with the bacterium *Rhizobium* sp. CRRU65 and those harvested from control plants influenced the antioxidant potential of the fruits. Oxidative stress in nematodes was induced by subjecting the animals to a temperature of 35 ^o^C that causes damage by the accumulation of reactive oxygen species (ROS)^21^.

**Fig. 2:**
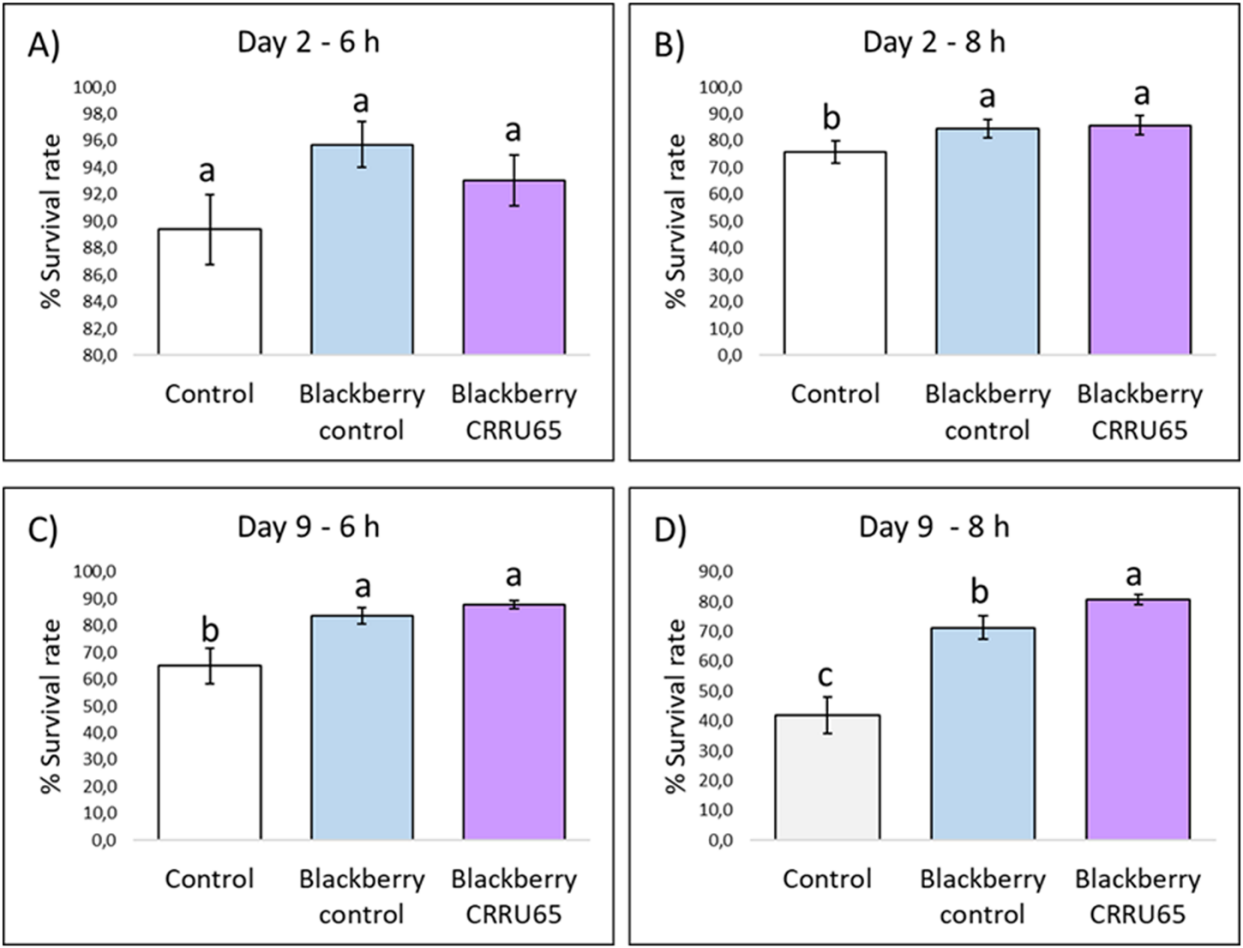
Survival rates of C. elegans exposed to different treatments and stress conditions. Percentages of survival following thermal stress (35 ^o^C, 6 h) applied at days 2nd (**A**) and 9th of adulthood (**C**) or following thermal stress (35 ^o^C, 8 h) applied at days 2nd (**B**) and 9th of adulthood (**D**) in N2 wild type *C. elegans* strain not treated (controls) and treated with BE. a–c letters indicate significant differences (*p*-value ≤ 0.05) among treatments according to Tukey test. Additionally, our results showed that exposure of nematodes to BE obtained from plants inoculated with the *Rhizobium* sp. CRRU65 strain significantly increased their survival rate in 9-day-old adult nematodes after 8 hours of stress, compared to tests conducted without BE and those with BE from uninoculated control plants. In these cases, the survival rate increased from 41.8% in trials without BE and from 71.4% in trials with BE from control plants to 80.7% (**D**). Our data suggest that the application of PGP bacteria exerts a dual probiotic effect, benefiting both the plant and the consumer by enhancing the nutraceutical properties of blackberries.

The biological activity of BE was evaluated in *C. elegans* as an in vivo model to determine whether differences in phenolic compound concentrations influence health. A thermal stress assay was conducted to compare the resistance of nematodes exposed to BE from inoculated and control plants. Also, negative control without BE was included. Data were taken on day 2 of adulthood in the *C. elegans* organism, after 6 and 8 hours of exposure to thermal stress, and on day 9 of adulthood, also after 6 and 8 h of exposure to thermal stress. In general terms, except on day 2 at 6 h, there were significant differences in the survival rate between nematodes growing on control with DMSO and the nematode exposed to the BE (Fig. 2A).

### Influence of BC in the expression of *daf-16, hsf-1*, *skn-1*, and *hsp-16* genes

Oxidative stress is regulated by several metabolic pathways in *C. elegans*. One of the most studied, and highly conserved in mammals, is the insulin/IGF-1 signaling (IIS) pathway The present study analyzes the influence of BE in the expression of the DAF-16, HSF-1, and SKN-1 transcriptional factors and the heat-shock proteins 16 (HPS*-*16).

**Fig. 3:**
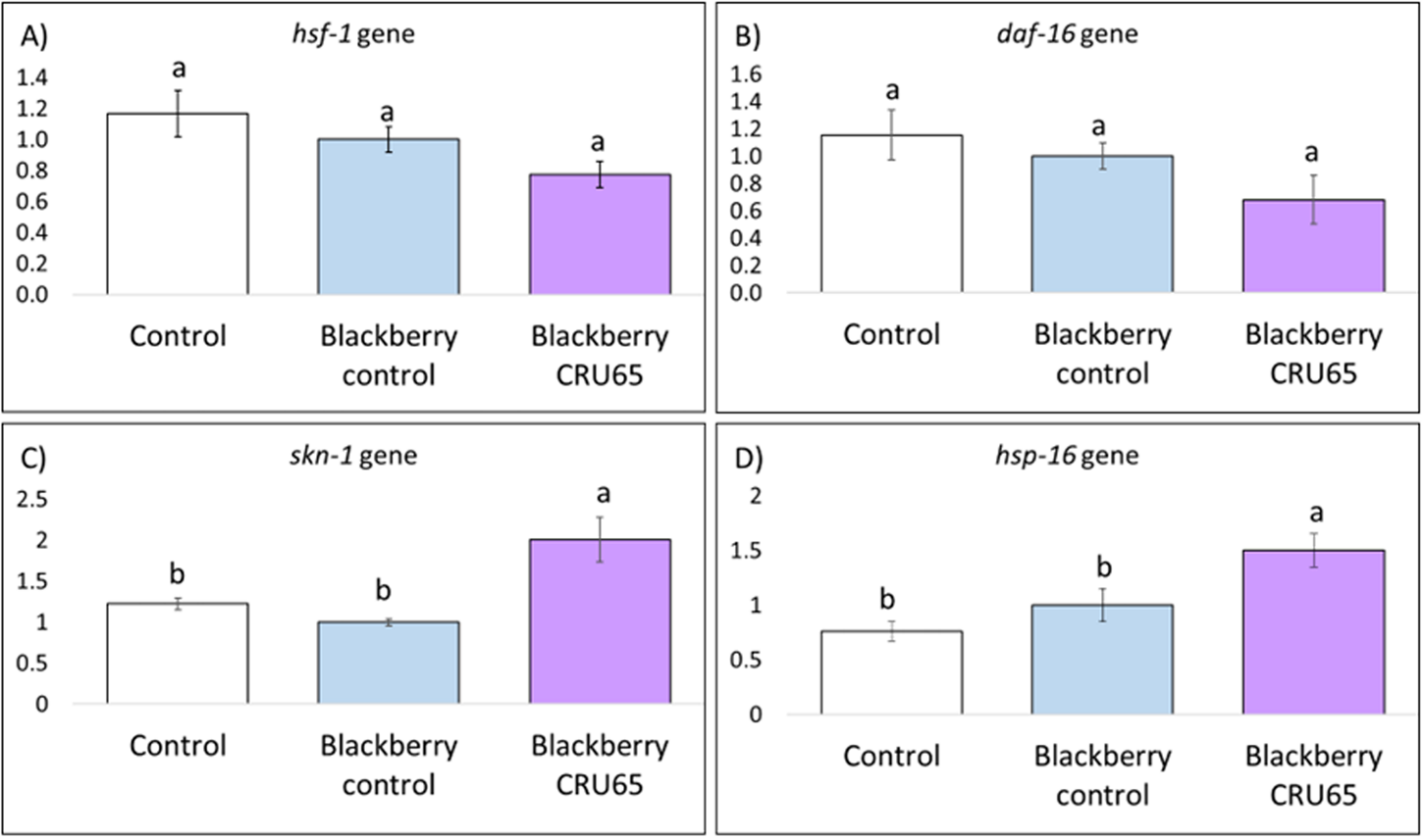
Analysis of gene expression in C. elegans exposed to different treatments and growth conditions. Relative gene expression of: **A** *hsf-1*; **B** *daf-16*; **C** *skn-1*; **D** *hsp-16* in model organism *C. elegans* growing on control plants, on plates made of blackberry extracts from non-inoculated plants (Blackberry control), and on plates made of blackberry extracts from *Rhizobium* sp. CRRU65 inoculated plants (Blackberry CRRU65). a–b letters indicate significant differences (*p*-value ≤ 0.05) among treatments according to Tukey test.

The expression of those genes was evaluated with and without BE. For all genes, the results showed that nematodes exposed to BE from uninoculated plants exhibited no significant differences compared to the control group. In contrast, exposure to BE from *Rhizobium* sp. CRRU65-inoculated plants led to a significant upregulation of *skn-1* and *hsp-16* genes, both associated with stress resistance and longevity (Fig. 3).

Therefore, the increased survival of nematodes to thermal stress observed in the previous section could be due to the increased expression of the *skn-1* and *hsp-16* genes, which could have been induced by the exposure of the nematode to the BE obtained from plants inoculated with *Rhizobium* sp. CRRU65. This hypothesis requires further investigation through targeted studies examining the expression of genes and metabolic pathways related to antioxidant activities.

## Discussion

Bacteria belonging to genus *Rhizobium* genus have been frequently studied in promoting plant growth in legume plants, mainly due to the production of IAA^22^. This effect has been documented and it has been observed that the inoculation of lettuce plants with the species *Rhizobium leguminosarum* increased the size of the aerial part of the plants or that the inoculation of tomato plants with the bacteria *Rhizobium calliandrae* LBP2-1, *Rhizobium mayense* NSJP1-1, and *Rhizobium jaguaris* SJP1-2 increased not only the growth of the plant, but also the quality of the fruit, thanks to the ability of these bacteria to solubilize phosphates, produce siderophores and synthesize IAA^4,23^. However, the application of these bacteria has gone further and extended to other types of crops^24,25^. In these systems, *Rhizobium* spp. were able not only to promote plant growth but also flowering and/or fruiting^3–6^. For instance, in edamame crops, the application of *Rhizobium* strains increased plant flowering. In mango trees, inoculation with *Rhizobium* sp. XXV increased the number of flowers and, consequently, the number of fruits, and in tomato plants inoculated with *Rhizobium calliandrae* LBP2-1, *Rhizobium mayense* NSJP1-1, and *Rhizobium jaguaris* SJP1-2 strains, it increased both the number and size of fruits^3–5^. All these effects were linked to an increase in the production of phytohormones, among which the synthesis of indoleacetic acid, a hormone related to flowering, stands out^26^. Similarly, inoculation of bacteria capable of solubilizing phosphate compounds from the soil has been shown to increase the number of flowers per plant and, in turn, the number of fruits in tomato plants^27^. In the in vitro tests carried out in this work, the bacterium *Rhizobium* sp. CRRU65 demonstrated the ability to solubilize both dicalcium phosphate and tricalcium phosphate, and to produce auxins, especially IAA. Therefore, the increase in blackberry fruit production observed after the application of the bacterium *Rhizobium laguerreae* CRRU65 could be related to these two activities.

For this work, we evaluated the effect of the endophytic *Rhizobium* CRRU65 strain on blackberry crops. Species identification is a basic requirement in microbiology, which is usually accomplished by 16S rRNA gene sequencing, since it is considered the reference taxonomic marker in prokaryotic organisms^28^. Although the 16S rRNA gene sequence comparison showed that CRRU65 strain had a similarity of 100% with respect to *Rhizobium laguerreae* FB206^T^, it has been described that multiple distinct species share identical 16S rRNA sequences within the genus *Rhizobium*^29^. For species differentiation, dDDH value was calculated, which allows the differentiation based on the genomes^30^. Values under 70% indicated that CRRU65 belonged to an undescribed species^31^. That is why, CRRU65 strain was identified as *Rhizobium* sp. CRRU65.Flowering is a process which is mainly influenced by photoperiod and vernalization, but in recent years, age, thermosensory, sugar, stress, nutrition, and hormonal signals have also been identified as factors which control floral transition^32^. In the in vitro tests carried out, *Rhizobium* sp. CRRU65 was able to produce auxins, especially IAA. Auxins can affect flowering and, specifically, IAA application on plants seemed to increase the number of flowers in crops such as *Antirrhinum majus* L., *Solanum lycopersicum* L., *Cucumis melo* L. or *Hibiscus sabdariffa* L.^33–36^. Moreover, in raspberry and strawberry crops, it has been reported that the over-expression of auxin-synthesis related gene (*iaaM*) resulted in the increment of the number of flowers per inflorescence and an increased number of inflorescences per plant, suggesting an important role for IAA^37^. Besides indole acetic acid, there are other plant hormones related to plant growth. Cytokinin is a key plant hormone involved in controlling the reproductive shoot architecture of flowering plants and boosting the size and number of most reproductive structures^38^. The genome of *Rhizobium* sp. CRRU65 contains genes involved in cytokinin production, such as *miaA*, *miaB*, *yvdD*, and *dapF*^39^. Putrescine, spermidine and spermine are polyamines related to a wide range of physiological and cellular processes, including growth, development and flowering^40,41^. The *speBC* and *gsp* genes were annotated in *Rhizobium* sp. CRRU65 genome. The *spe* system is involved in putrescine biosynthesis while the *gsp* gene is a glutathionyl spermidine synthetase involved in spermidine synthesis^42,43^. Putrescine also showed to increase fruit quality by inhibited ethylene production and improve antioxidant enzyme activity during fruit ripening^44^.

Additionally, nutrition plays a critical role in plant flowering^32^. The elements with the greatest impact are nitrogen, phosphorus, and potassium. Studies have shown that limitations in the availability of these three nutrients reduce plant flowering^45^. In vitro, *Rhizobium* sp. CRRU65 demonstrated to be able to solubilize Ca_2_PO_3_ and AlKO_6_Si_2_. In that sense, those activities could be related to the number of flowers increment which was observed during greenhouse assay. Vitamins are also essential compounds for the correct development of plants. B vitamins include vitamin B1 (thiamine and its derivatives), vitamin B2 (riboflavin), vitamin B3 (niacin, nicotinamide and nicotinamide riboside), vitamin B5 (pantothenate), vitamin B6 (pyridoxine, pyridoxal, pyridoxamine and derivatives), vitamin B7 (biotin), vitamin B9 (folates and their derivatives) and vitamin B12 (cobalamin). In plants, these vitamins are involved in multiple processes where they act as cofactors, coenzymes, they also participate in redox reactions and reduce oxidative damage^46^. Various genes related to the synthesis of these vitamins were annotated in CRRU65 genome such as *thiDEFKLMNO*, *cobACFGLNPQRSTU*, *pdxAHJK*, *ribABDEFH*, *panBCE*, and *folABCDEKP*. These genes encode for vitamin B1 thiamine, B12 cobalamin, B6 pyridoxine, B2 riboflavin, B5 pantothenate and B9 folate biosynthesis, respectively^47–50^.

In vitro, in silico, and in vivo analyses showed that the endophytic bacteria *Rhizobium* sp. CRRU65 could have a greater effect on crop development, influencing flowering and fruit production. The effect of the bacteria inoculation on blackberry fruit production was evaluated under greenhouse conditions, since this crop is usually grown in protected cultivation^51^. The evaluated parameters were chlorophyll content, number of flowers per plant, number of fruits per plant, average fruit weight per plant, and total fruit weight per plant. The subsequent in vivo assay showed the conservation of chlorophyll content and the increment of flowers and fruits in *Rhizobium* sp. CRRU65 inoculated plants. Regarding the first parameter, this effect was previously studied in plants such as mango, strawberry or pistachio. In these crops, there were alterations in the content of different compounds, including chlorophyll, before fruit production began^52–54^. Our results showed that inoculation did not affect chlorophyll content, suggesting that the treatment with the selected bacteria does not significantly impact the photosynthetic capacity of the plants. Additionally, *Rhizobium* sp. CRRU65 inoculation of blackberry plants demonstrated an increase in total fruit production, primarily attributed to an increment in the number of flowers. Previous studies reported that *Rhizobium* spp. inoculation enhanced both crop yields and quality^4,6^. In recent years, the demand for foods with nutraceutical properties has increased considerably^55^. Blackberries are included among fruits with great antioxidant potential, which is derived from the high content of phenolic compounds^10,12,56,57^. Inoculation of blackberry crops with growth-promoting bacteria could alter this composition and thus affect their antioxidant capacities^58^. The analysis of the collected blackberries showed that the bacteria *Rhizobium* sp. CRRU65 inoculation was not only capable of increasing fruit production, but also modified the concentration of their phenolic compounds, specifically increasing the concentration of the flavonoids cyanidin-3-O-glucoside and kaempferol acetylhexoside, and the tannins sanguiin H6 and trigalloyl HHDP glucoside. Similarly, inoculation of strawberry with *Rhizobium laguerreae* PEPV16 under greenhouse and field conditions induced an increase in the concentration of epicatechin, p-coumaric acid and ellagic acid^7,59^. In this way, inoculation of blueberry crops with *Rhizobium laguerreae* PEPV16 increased the concentration of polyphenols in the fruits such as quercetin 3-O-rutinoside and delphinidin 3-O-arabinoside, or inoculation of lettuce plants with *Rhizobium laguerreae* increases the concentration of phenolic compounds in the leaves like caffeoyl acids derivates. Additionally, inoculation of lettuce plants with another *Rhizobium* sp., *Rhizobium* sp. GPTR29, resulted on the increment of apigenin derivative and caffeoyl tartaric acid under normal conditions, but also the increment of total flavonoid content under salinity conditions (100 mM NaCl)^2,6,23^. Among flavonoids, anthocyanins are one of the most important compounds in blackberries composition, responsible for the dark blue color and for their bioactive properties, associated with a reduction in insulin resistance, a decreased risk of myocardial infarction and moderating the inflammatory response^60^. Cyanidin 3-*O*-glucoside is the main anthocyanin in blackberries, and exhibit significant antioxidant, antidiabetic, anti-inflammatory and cytoprotective effects against various oxidative stress-induced disorders^61^. Another well-known flavonoid found in blackberries is kaempferol, which possess anticarcinogenic and anti-inflammatory effects^62^. Regarding tannins in the blackberries composition, sanguiin H6 is one of the most abundant, which has been related to antimicrobial, antiviral, anticancer, anti-inflammatory and osteoclastogenesis inhibitory activities^17^. Trigalloyl HHDP glucoside and glucogallin are also tannins that have been, to a lesser extent, associated with the health benefits of blackberry compsumtion^18^. Although phenolic compounds are usually related with health promotion, it has been reported that most health benefits of blackberries are due to the bioactivity of anthocyanins^10^. Since *Rhizobium* sp. CRRU65 inoculation significantly increased the concentration of these compounds, we hypothesize that these compositional changes could modify the antioxidant activity linked to blackberry consumption.

Phenolic compounds contribute to the high antioxidant power of blackberries and some studies suggest that their consumption may reduce the risk of obesity, cardiovascular diseases, degenerative diseases, and various forms of cancer^19^. For instance, the intake of anthocyanins has been associated with a reduction in insulin resistance, a lower risk of myocardial infarction and a moderation of the inflammatory response^20^. In this regard, we conducted an assay using the model organism *C. elegans* to assess whether the differences observed in phenolic compounds concentration between blackberries harvested from plants inoculated with the bacterium *Rhizobium* sp. CRRU65 and those harvested from control plants influenced the antioxidant potential of the fruits. Oxidative stress in nematodes was induced by subjecting the animals to a temperature of 35 ^o^C that causes damage by the accumulation of reactive oxygen species (ROS)^21^. *C. elegans* has been used for years as a biological model for clinical studies related to humans, including the study of diseases such as Alzheimer’s and Parkinson’s and conditions such as obesity or aging^14^. In all these processes, a key factor is the accumulation of reactive oxygen species, which increases cellular damage and accelerates or induces these diseases^63,64^. Antioxidant compounds help reduce oxidative damage and prevent both aging and the diseases derived from it^10,28,65^. Temperature is one of the critical environmental conditions in the life of *C. elegans* and its increment increases oxidative damage in the organism, which is induced by the accumulation of ROS. That is why thermal tolerance assays are performed to evaluate oxidative stress in the nematode^66^. In this work, the nematodes exposition to blackberries collected from *Rhizobium* sp. CRRU65 inoculated plants proved to significantly increase survival under oxidative stress conditions, showing for the first time the promising effect on enhancing antioxidant activity of the fruits after PGP bacteria inoculation. This result could be related to the increment in the concentration of phenolic compounds such as cyanidin-3-o-glucoside which was described as one of the main ones responsible for the blackberry antioxidant activity^67–69^.

Oxidative stress is regulated by several metabolic pathways in *C. elegans*. One of the most studied, and highly conserved in mammals, is the insulin/IGF-1 signaling (IIS) pathway^70^. This pathway regulates the nuclear entry of the transcription factors DAF-16 (ortholog of FoxO in humans), HSF-1 (ortholog of human HSF1 (heat shock transcription factor 1), and SKN-1 (ortholog of Nrf in humans)^13^. Inside the nucleus, these transcription factors regulate the expression of various genes involved in oxidative stress mitigation, such as superoxide dismutases, which convert the superoxide anion into hydrogen peroxide; catalases, which convert hydrogen peroxide into water; or glutathione S-transferases, which are involved in the detoxification of endogenous forms of ROS^64,71^. Additionally, the transcription factor HSF-1 activates the expression of genes encoding molecular chaperones, such as *hsp-16* and *hsp-70*, involved in the longevity and thermotolerance of *C. elegans*^72^. The present study analyzes the influence of BE in the expression of the DAF-16, HSF-1, and SKN-1 transcriptional factors and the heat-shock proteins 16 (HPS*-*16), since they plays a key role in thermotolerance^73^. Our results showed that the treatment blackberries collected from plants inoculated with the *Rhizobium* sp. CRRU65 upregulated the expression of *skn-1* and *hsp-16* genes in the nematodes. Increased expression of these genes was associated with reduced oxidative stress^74,75^. On one hand, transcription factor SKN-1 is a crucial player in the responses to oxidative stress whose overexpression increases resistance to oxidative stress and longevity^72^. Although this transcription factor is usually bonded to DAF-16 and HSF-1, it have been reported that nematodes exposition to some compounds such as rose essential oil or mulberry anthocyanins can promote survival under oxidative stress through *skn-1* overexpression, but not *daf-16* or *hsf-1*^60,72^. On the other hand, heat shock proteins function as molecular chaperones and proteases, preventing the accumulation of aggregated proteins in response to heat and other stressors. This protective role helps to inhibit the aggregation of oxidized or damaged proteins, allowing for their refolding or degradation before they become cytotoxic^66^. In the present study, our results indicate that BE increased *hsp-16* expression in *C. elegans*, which may contribute to the observed increment in heat stress resistance.

Oxidative stress in *C. elegans* involves multiple interconnected mechanisms. Therefore, the effects observed in this study may result from modifications in other pathways that were not examined. Further investigation into additional metabolic pathways would be necessary to gain a deeper understanding of the effects on the nematode resulting from alterations in the composition of phenolic compounds in fruits, induced by *Rhizobium* sp. CRRU65 inoculation. In conclusion, results obtained in this work confirm *Rhizobium* sp. CRRU65 could be a candidate for the development of a biostimulant for crop improvement. Inoculation of blackberry plants with this bacterium increased both fruit production and the phenolic content of the fruits, which seemed to impact on their health properties. This effect was confirmed by exposing the model organism *C. elegans* BE collected from inoculated plants. In the tests, we observed a survival rate increment under conditions of oxidative stress linked to an increase in the expression of some genes involved in the metabolic pathways of stress tolerance. In this sense, this work highlights the benefit of using endophytic bacteria in agricultural crops, which not only represents an advantage for farmers, who would obtain more efficient crops, but also an advantage for consumers, since they would obtain fruits of higher nutraceutical quality.

## Methods

### Bacterial strain and 16S gene identification

The CRRU65 strain was isolated from the inside of surface-sterilized roots of *Rubus ulmifolius* Schott grown in a soil in Ciudad Rodrigo, Salamanca, Spain (40° 35′ 23.4″ N 6° 29′ 53.6″ W), using the standard method of Vincent (1970) on yeast mannitol agar (YMA) plates at 28 °C.

The amplification and sequencing of the 16S rRNA gene were carried out as indicated previously Rivas et al.^76^ using the primers 27 F (5′-AGAGTTTGATCCTGGCTCAG-3′) and 1522 R (5′ AAGGAGGTGATCCANCCRCA 3′). Sequences were obtained by Sanger using the same primers as those for amplification. Generated fragments from the same sequence were assembled using the SeqMan II program. The assembled sequence was compared with those from GenBank using the BLASTN program, selecting “type strains” as phylum to perform the search^77^. The obtained sequence was subsequently deposited to GenBank (accession number: OQ196016).

### Evaluation of in vitro PGPs activities

In vitro PGP activities were evaluated from a bacterial suspension made of pure culture of the bacteria grown for 5 days at 28 °C in YMA medium in sterile distilled water at an approximate density of 1·10^6^ CFU/mL according to the McFarland scale.

Inorganic phosphate solubilization was studied by using Pikovskaya medium (0.5 g/L of yeast extract, 10 g/L of glucose, 5 g/L of Ca_2_PO_3_ or Ca_3_(PO_3_)_2_, 0.5 g/L of (NH_4_)_2_SO_4_, 0.2 g/L of KCl, 0.1 g/L of MgSO_4_, 0.0001 g/L of MnSO_4_, 0.0001 g/L of FeSO_4_, and 20 g/L of agar)^78^ and NBRIP medium (National Botanical Research Institute’s Phosphate growth medium) (10 g of glucose, 0.1 g/L of (NH_4_)_2_SO_4_, 0.2 g/L of KCl, 0.25 g/L of MgSO_4_ · 7 H_2_O, 5 g/L of Ca_5_(PO_4_)_3_(OH) and 20 g/L of agar)^79^. Plates were inoculated with 5 μL of the bacterial suspension and incubated for 5 days at 28 °C. Positive results were observed as a transparent halo surrounding the colony.

Potassium compound solubilization was studied using Aleksandrov medium (0.5 g/L of MgSO_4_ · 7 H_2_O, 0.1 g/L of CaCO₃, 2 g/L of AlKO_6_Si_2_, 5 g/L of glucose, 0.005 g/L of FeCl_3_, 2 g/L of Ca_3_(PO_4_)_2_ and 20 g/L of agar)^80^. Plates were inoculated with 5 μL of the bacterial suspension and incubated for 5 days at 28 °C. Positive results were observed as a transparent halo surrounding the colony.

Siderophores production was performed by using M9-chrome azurol S (CAS) agar medium, which was prepared from four different solutions, sterilized individually and subsequently mixed^81^. Solution 1:10 mL of FeCl_3_ · 6 H_2_O (1 mM) dissolved in HCl (10 mM), with an aqueous solution of 50 mL of CAS (1.21 mg/mL). The resulting solution was mixed with 40 mL of an aqueous solution of HDTMA (1.82 mg/mL). Solution 2: 30.24 g/L of piperazine-N,N’-bis[2-ethanesulfonic acid], 0.3 g/L of KH_2_PO_4_, 0.5 g/L of NaCl, 1 g/L of NH_4_Cl, and 15 g/L of agar. Solution 3: 2 g of sucrose, 2 g of mannitol, 493 mg of MgSO_4_ · 7 H_2_O, 11 mg of CaCl_2_, 1.17 mg of MnSO_4_ · H_2_O, 1.4 mg of H_3_BO_3_, 0.04 mg of CuSO_4_· 5H_2_O, 1.2 mg of ZnSO_4_·7 H_2_O, 1 mg of Na_2_MoO_4_ · 2 H_2_O, and 70 mL of H_2_O. Solution 4: 30 mL of cas-amino acids to 10% (w/v), sterilized by filtration with a 0.22 μm membrane. Once all the solutions were prepared, they were sterilized in an autoclave. Subsequently, when the temperature of the medium had dropped to 50 °C, the solutions were mixed, with solution 4 being the last to be added. The plates were inoculated with 5 μL of bacterial suspension. Positive results were observed as an orangish halo surrounding the colony.

The presumptive ability to produce auxin was evaluated by using John Howieson minimal medium (JMM medium)^82^ (1.8 g/L of D-(+)-galactose, 1.5 g/L of L-(+)-arabinose, 0.147 g/L of pyruvate, 0.0261 g/L of K_2_HPO_4_, 0.0055 g/L of FeSO_4_ · 2 H_2_O, 0.147 g/L of CaCl_2_ · 2 H_2_O, and 1 mL/L of vitamin solution (20 mg/L of riboflavin, 20 mg/L of P-amino benzoic acid, 20 mg/L of nicotinic acid, 20 mg/L of biotin, 20 mg/L of thiamine-HCl, 20 mg/L of pyridoxine-HCl, 20 mg/L of calcium pantothenate, and 120 mg/L of inositol; dissolved in Na_2_HPO_4_ at 0.05 M (pH 7.0) and sterilized by filtration with a 0.22 μm membrane). All ingredients were mixed, dispensed into vials (3 mL per vial) and autoclaved, except for the vitamin solution which was prepared separately. The media were supplemented with tryptophan, at a final concentration of 0.165 g/L. A 3 μL aliquot of the bacterial suspension was inoculated into the media and incubated at 28 °C for 72 hours in the dark. After incubation, 1.5 mL from each vial was transferred to an Eppendorf-type tube and centrifuged at 14,000 r.p.m. for 1 min. Next, 1 mL of the supernatant was collected and added to the spectrophotometry measurement tube, to which 500 μL of the Salkowski reagent (20 mL of FeCl_3_ (0,5 M) and 980 mL HClO_4_ (35% v/v)) was added, mixing all by pipetting. Color intensity was measured using an ATI Unicam 8625 absorption spectrophotometer (Mattson^TM^, USA) at a wavelength of 550 nm. The auxin concentration was extrapolated from the absorbance obtained using the standard line: y = 2.4637 · x + 0.0053 (where: y = Absorbance, x = Concentration in mg/L). Additionally, the production of 3-indoleacetic acid (IAA) was also determined by high-performance liquid chromatography (HPLC) from the growth obtained in the JMM media after filtration with a 0.22 μm membrane. The measurement was carried out at the Elemental Analysis, Chromatography and Mass Service of the University of Salamanca^83^.

### Genome evaluation

Genomic DNA was extracted by using the “ZR Fungal/Bacterial DNA MiniPrep” kit (Zymo Research™) following the manufacturer’s instructions and preserved at −20 °C. Sequencing was carried out using the Illumina Miseq platform (2x250bp). The sequences obtained were assembled using Velvet 1.2.10 based on Linux^84^ and annotation was carried out using the RAST 4.0 program (http://rast.theseed.org), which allows automatic annotation of the genome sequences obtained^85^. The Benchmarking Universal Single-Copy Orthologs (BUSCO) Version 5.5.0 software was used to evaluate the completeness of the assembled genomes. The annotated genome was also analyzed with the online tools PLaBAse, which searches for genes related to plant-microorganism interactions^86^. The draft genome sequence was deposited in DDBJ/EMBL/GenBank under the Bioproject PRJNA1222147 (accession number JBLYZH000000000). Digital DNA–DNA hybridization (dDDH) values were calculated using the Genome-to-Genome Distance Calculator website service from DSMZ (GGDC 2.1)^31^.

### Blackberry production assay on greenhouse conditions

*Rubus fructicosus* var. Asterina plants, approximately 60 cm in height, were purchased from the nursery “Arándanos el Cierrón” (Les Vegues, 33311 Fuentes, Asturias, Spain). The acquired plants were transplanted into 5 L pots with soil and were maintained in a greenhouse under constant humidity and temperature conditions throughout the trial. Inoculation was carried out at the beginning of the reproductive phase, when spring new shoots began to emerge. For inoculation, bacterial suspensions were prepared at a concentration of 10^8^ CFU/mL and 10 mL were inoculated into each plant. A total of 20 plants per treatment were used and randomly distributed in the greenhouse. A negative control was arranged with the same number of plants, applying 10 mL of sterile distilled water. Trials continued till the end of the harvesting period. After this time, the number of fruits per plant and treatment and the fruits’ weight were measured. Chlorophyll was also quantified by using a Chlorophyll meter (Konica Minolta SPAD-502 PLUS). Statistical analysis of the data was performed by one-way analysis of variance (ANOVA), based on the Tukey HSD test (*p* ≤ 0.05), using the statistical software for PC Rstudio (version 4.1.2).

### Quantitative analysis of phenolic compounds

For each treatment, extractions were performed in triplicate employing 500 mg of lyophilized and powdered fruits. Briefly, phenolic compounds were extracted using 150 mL of acidified MeOH:H_2_O (80:20). The supernatant was recovered by filtration with Whatmann paper (0,22 um), extraction was performed three times, and the corresponding supernatants were combined and concentrated in rotavapor. The final volume was adjusted to 10 mL with MiliQ water. The obtained extracts were analyzed by HPLC–DAD–MS to determine the phenolic composition. Briefly, a Hewlett–Packard 1100 series liquid chromatograph (Agilent Technologies, Waldbronn, Germany), equipped with a C18 reversed-phase column (5 μm, 150 mm × 4.6 mm) (Aqua, Phenomenex, Torrance, CA) thermostated at 35 °C, was employed. The conditions employed were established based on previous studies on phenolic composition in food matrices^87^, as follows: the mobile phase was composed of solvent A, 0.1% (*v*/*v*) TFA aqueous solution, and solvent B, HPLC grade acetonitrile. The following elution profile was used at a flow rate of 0.5 mL min^−1^: isocratic at 90% A for 5 min, from 90% to 85% A for 15 min, isocratic at 85% A for 5 min, from 85% to 82% A for 5 min and from 82% to 65% A for 20 min. Chromatograms were recorded at 280, 330, 360 and 520 nm as preferred wavelengths. The 3200 Qtrap mass spectrometer (Applied Biosystems, Darmstadt, Germany) was connected to the HPLC system via the DAD cell outlet and spectra were recorded in positive ion mode using the same conditions as described previously^88^. The identification of the compounds was performed based on retention times, UV-vis spectra and mass spectra, comparing them with commercial standards, when available. Individual compounds were quantified using peak area values obtained from chromatograms recorded at 280 nm (protocatechuic acid glucoside or ellagic acid), 330 nm (caffeic acid derivatives), 360 nm (flavone and flavonol derivatives), or 520 nm (anthocyanins), applying the external standard method as follows: chlorogenic acid (5-caffeoylquinic acid) was quantified using its own calibration curve; procyanidin B dimer and epicatechin were quantified using the epicatechin calibration curve; ellagic acid, ellagic acid pentoside and ellagitannins (trigalloyl HHDP glucose, sanguiin H6 isomer and sanguiin H6) were determined as ellagic acid equivalents; eriodictyol hexoside, glucogallin and unknown compounds were quantified as gallic acid equivalents; flavonols (kaempferol acetylhexoside, kaempferol rutinoside, quercetin glucuronide, quercetin hexoside, rutin, quercetin acetylhexoside and quercetin pentoside) were expressed as quercetin glucuronide equivalents; and anthocyanins (cyanidin 3-*O*-glucoside, cyanidin 3-*O*-pentoside, cyanidin 3-*O*-malonylglucoside, cyanidin 3-*O*-pentoside and pelargonidin 3-*O*-glucoside) were determined as cyanidin 3-*O*-glucoside equivalents. The results were obtained from the mean value of three independent analyses and were expressed in g/kg of dry fruit weight.

### In vivo assays on *C. elegans* model

*C. elegans* wild type strain N2 was obtained from the *Caenorhabditis* Genetics Centre (CGC) at the University of Minnesota (Minneapolis, MN, USA). Nematode Growth Medium (NGM) (Table 2) inoculated with *Escherichia coli* OP50 was used for nematodes maintenance. Synchronization of worm cultures was achieved by allowing gravid hermaphrodites to lay eggs for 2–3 h on fresh plates. The eggs obtained during synchronization were placed in either control plates or plates containing blackberry extracts (BE).

**Table 2:** Composition of the NGM medium

NGM (Nematode growth medium)	
NaCl	1.2 g
Peptone	1 g
CaCl_2_ 1 M pH 6,0	400 µL
Cholesterol (5 mg/mL ethanol)	400 µL
MgSO_4_ 1 M *	400 µL
Buffer phosphate 1 M pH 6,0	400 µL
Sodium ampicilin (50 µg/mL)	400 µL
Nistatin (1 g/100 mL in ethanol: ammonium acetate 1:1)	700 µL
Agar	6,8 g
H_2_O	390 mL

Components marked with an asterisk are sterilized by filtration and added after autoclaving the medium.

Blackberry extracts (BE) were dissolved in dimethyl sulfoxide (DMSO) and added to the NGM in a final concentration of 200 µg of BE/mL. Control plates contained DMSO at the same final concentration (0.1% DMSO, v/v).

Oxidative stress in nematodes was induced by subjecting them to 35 °C^21^. Nematodes were born on NGM agar plates (Ø 100 mm) containing either the BE or 0.1% of DMSO and they were cultivated at 20 °C until they reached adulthood. At that moment, they were transferred to new plates containing either the BE or 0.1% of DMSO, but also containing fluorodeoxyuridine (FUdR) at a concentration of 150 µM, which prevents reproduction and progeny overgrowth. Every two days, nematodes were transferred again to fresh plates also containing FUdR and different treatments until the measurement day. Then, they were transferred with a platinum wire to agar plates (Ø 35 mm, 20 nematodes per plate) and then switched to 35 °C for 6 h in an incubator. After that time, the dead and alive nematodes were counted. Next, the same nematodes were exposed to 2 more hours at 35 °C, and dead and alive nematodes were again counted. Assays were performed with approximately 100 nematodes per treatment. Three independent experiments were performed for each extract and conditions were assayed. Statistical differences between treatments were obtained by constructing contingency tables. Statistical significance was obtained using the Chi Square Test. In each analysis, differences were considered statistically significant at the *p* < 0.05 level.

For gene expression analysis, nematodes were maintained on NGM agar plates (Ø 100 mm) containing either the 200 µM BE or 0.1% of DMSO for 5 days at 20 °C. After that, nematodes were collected with M9 buffer, centrifuged at 10,000 g for 1 min, the pellet was resuspended in 300 µL of M9, and 3.5 µL of 2-mercaptoethanol was added. ZymoBIOMICS RNA Miniprep Kit was used for total RNA extraction, and the homogenization step was carried out with FastPrep-24 TM 5 G (Thermo Scientific™), using a program of seven pulses of 10 s at 5.5 m/second, with a 20 s rest between pulses. Extracted RNA was quantified with Qubit RNA HS (Thermo Scientific™), treated with DNAse to eliminate undesirable DNA residues by using the TURBO DNA-free^TM^ Kit (Thermo Scientific™) and again quantified with Qubit RNA HS (Thermo Fisher Scientific, Waltham MA, USA). Finally, to obtain cDNA, the First Strand cDNA Synthesis Kit (Thermo Scientific™) was used, being quantified with Qubit dsDNA HS (Thermo Scientific™) at the end.

The expression of mRNA was assessed by quantitative real-time PCR, using SYBR green as the detection method. Gene expression data were analyzed using the comparative 2^-∆∆CT^ method with *act-1* as the normalizer. Nine independent experiments were performed; the dissociation curve was determined to confirm a single amplification. The used primers are described in Table 3.

**Table 3:** List of primers used for *Caenorhabditis elegans* gene expression analysis

Name	Gene	Sequence (5’ – 3’)*
*act-1-F*	Actin	CCAGGAATTGCTGATCGTATG
*act-1-R*		GGAGAGGGAAGCGAGGATAG
*skn-1-F*	Skinhead	AGTGTCGGCGTTCCAGATTTC
*skn-1-R*		GTCGACGAATCTTGCGAATCA
*daf-16-F*	abnormal dauer formation	CCAGACGGAAGGCTTAAACT
*daf-16-R*		ATTCGCATGAAACGAGAATG
*hsf-1-F*	Heat-shock factor	GAAATGTTTTGCCGCATTTT
*hsf-1-R*		CCTTGGGACAGTGGAGTCAT
*hsp-16-F*	Heat-shock protein	CTGCAGAATCTCTCCATCTGAGTC
*hsp-16-R*		AGATTCGAAGCAACTG-CACC

*Primers obtained from Gutierrez-Zetina et al.^73^.

Quantitative PCR (RT-qPCR) was performed in a StepOnePlusTM Real-Time PCR System thermocycler (Applied Biosystems) using PowerTrack SYBR® Green 2X Mater Mix (Applied Biosystems) as fluorochrome. Primers were added at 10 μM. The PCR conditions for a 10 μL reaction were 20 s at 95 °C for the holding stage; followed by 40 cycles of 3 s at 95 °C and 30 s at 60 °C for the cycling stage; and 15 s at 95 °C, 1 min at 60 °C, 15 s at 95 °C for the melting curve stage.

## Supplementary information

Supplementary Material.

## Data Availability

The 16S rRNA gene and genome sequences were deposited at DDBJ/EMBL/GenBank under accession numbers OQ196016 and JBLYZH000000000, respectively.

## References

[1] Umesha, S., Singh, P. K. & Singh, R. P. Microbial biotechnology and sustainable agriculture. In *Biotechnology for Sustainable Agriculture: Emerging Approaches and Strategies* (eds Singh, R. L. & Mondal, S.), pp. 185–205 (Elsevier, 2017).

[2] Ayuso-Calles, M. et al. *Rhizobium laguerreae* improves productivity and phenolic compound content of lettuce (*Lactuca sativa* L.) under saline stress conditions. *Foods* **9**, 1166 (2020). 32847018 10.3390/foods9091166 PMC7555320

[3] Santos-Villalobos, D. S. et al. Growth promotion and flowering induction in mango (*Mangifera indica* L. cv ‘Ataulfo’) trees by *Burkholderia* and *Rhizobium* inoculation: morphometric, biochemical, and molecular events. *J. Plant Growth Regul.* **32**, 615–627 (2013).

[4] Gen-Jiménez, A. et al. Enhance of tomato production and induction of changes on the organic profile mediated by *Rhizobium* biofortification. *Front. Microbiol.* **14**, 1235930 (2023). 37601341 10.3389/fmicb.2023.1235930 PMC10433389

[5] Lestari, T., Apriyadi, R. & Amandha, G. Growth and yield of edamame soybean in post-tin mining land with application of *Rhizobium* bacteria and organic fertilizer. *IOP Conf. Ser. Earth Environ. Sci.* **694**, 012038 (2021).

[6] Flores-Félix, J. D. et al. Differential response of blueberry to the application of bacterial inoculants to improve yield, organoleptic qualities and concentration of bioactive compounds. *Microbiol. Res.* **278**, 127544 (2024). 37988818 10.1016/j.micres.2023.127544

[7] Flores-Félix, J. D. et al. *Rhizobium* and *Phyllobacterium* bacterial inoculants increase bioactive compounds and quality of strawberries cultivated in field conditions. *Food Res. Int.* **111**, 416–422 (2018). 30007704 10.1016/j.foodres.2018.05.059

[8] Nataraj, B. H., Ali, S. A., Behare, P. V. & Yadav, H. Postbiotics-parabiotics: the new horizons in microbial biotherapy and functional foods. *Microb. Cell Fact.* **19**, 1–22 (2020). 32819443 10.1186/s12934-020-01426-w PMC7441679

[9] Brennan, R. M. et al. Berry crops. *Hortic. Plants People Places* **1**, 301–325 (2014).

[10] Robinson, J. A., Bierwirth, J. E., Greenspan, P. & Pegg, R. B. Blackberry polyphenols: review of composition, quantity, and health impacts from in vitro and in vivo studies. *J. Food Bioact.* **9**, 40–51 (2020).

[11] Bhuyan, B. & Dutta, A. A review on the phytochemical, pharmacological and traditional profile on the *Rubus* genus in north-eastern and western parts of India. *Curr. Trends Pharm. Res*. **8**, 1 (2021).

[12] Meng, Q., Manghwar, H. & Hu, W. Study on supergenus *Rubus* L.: edible, medicinal, and phylogenetic characterization. *Plants* **11**, 1211 (2022). 10.3390/plants11091211 PMC9102695 35567211

[13] Ayuda-Durán, B., González-Manzano, S., González-Paramás, A. M. & Santos-Buelga, C. *Caenorhabditis elegans* as a model organism to evaluate the antioxidant effects of phytochemicals. *Molecules* **25**, 3194 (2020). 10.3390/molecules25143194 PMC7397024 32668705

[14] Markaki, M. & Tavernarakis, N. *Caenorhabditis elegans* as a model system for human diseases. *Curr. Opin. Biotechnol.* **63**, 118–125 (2020). 31951916 10.1016/j.copbio.2019.12.011

[15] Zhang, S., Li, F., Zhou, T., Wang, G. & Li, Z. *Caenorhabditis elegans* as a useful model for studying aging mutations. *Front. Endocrinol*. **11**, 554994 (2020). 10.3389/fendo.2020.554994 PMC7570440 33123086

[16] Hidalgo, G. I. & Almajano, M. P. Red fruits: extraction of antioxidants, phenolic content, and radical scavenging determination: a review. *Antioxidants* **6**, 7 (2017). 10.3390/antiox6010007 PMC5384171 28106822

[17] Gesek, J., Jakimiuk, K., Atanasov, A. G. & Tomczyk, M. Sanguiins—promising molecules with broad biological potential. *Int. J. Mol. Sci.* **22**, 12972 (2021). 34884795 10.3390/ijms222312972 PMC8657505

[18] Mal, S. & Pal, D. Tannins and polyphenols extracted from natural plants and their versatile application. *Adv. Struct. Mater.* **140**, 715–757 (2021).

[19] Mikulic-Petkovsek, M. et al. Fruit quality characteristics and biochemical composition of fully ripe blackberries harvested at different times. *Foods* **10**, 1581 (2021). 34359449 10.3390/foods10071581 PMC8304799

[20] Ruddock, P. L., Facey, P., Sieniawska, E. & Baj, T. T. *Pharmacognosy: Fundamentals, Applications and Strategies,* 2nd edn. (eds McCreath, S. B. & Clement, Y. N.), pp. 211–251 (Academic Press, 2024).

[21] Ayuda-Durán, B. et al. Antioxidant characterization and biological effects of grape pomace extracts supplementation in *Caenorhabditis elegans*. *Foods* **8**, 75 (2019). 10.3390/foods8020075 PMC6406641 30781355

[22] Dashadi, M., Khosravi, H., Moezzi, A. & Nadian, H. Co-Inoculation of *Rhizobium* and *Azotobacter* on growth indices of faba bean under water stress in the green house condition. *Adv. Stud. Biol.* **3**, 373–385 (2011).

[23] Ayuso-Calles, M. et al. Effect of *Rhizobium* mechanisms in improving tolerance to saline stress in lettuce plants. *Chem. Biol. Technol. Agric*. **10**, 89 (2023).

[24] Etesami, H., Etesami, H., Alikhani, H. A. & Akbari, A. A. Evaluation of plant growth hormones production (IAA) ability by Iranian soils rhizobial strains and effects of superior strains application on wheat growth indexes. *World Appl. Sci. J.* **6**, 1576–1584 (2009).

[25] Purwaningsih, S., Dewi, T. K., Sutisna, E. & Nugroho, A. A. Effectiveness of *Rhizobium* bacteria as biofertilizer on the growth of soybean (*Glycine max* L) in the greenhouse. *AIP Conf. Proc*. 2973 (2024).

[26] Fahde, S., Boughribil, S., Sijilmassi, B. & Amri, A. Rhizobia: a promising source of plant growth-promoting molecules and their non-legume interactions: examining applications and mechanisms. *Agriculture* **13**, 1279 (2023).

[27] Haile, D., Tesfaye, B. & Assefa, F. Tomato production under synergistic application of phosphate solubilizing bacteria and phosphate amendments. *Adv. Agric*. **2023**, 4717693 (2023).

[28] Shukitt-Hale, B., Carey, A. N., Jenkins, D., Rabin, B. M. & Joseph, J. A. Beneficial effects of fruit extracts on neuronal function and behavior in a rodent model of accelerated aging. *Neurobiol. Aging* **28**, 1187–1194 (2007). 16837106 10.1016/j.neurobiolaging.2006.05.031

[29] Young, J. P. W., Jorrin, B., Moeskjær, S. & James, E. K. *Rhizobium brockwellii* sp. nov., *Rhizobium johnstonii* sp. nov. and *Rhizobium beringeri* sp. nov., three genospecies within the *Rhizobium leguminosarum* species complex. *Int. J. Syst. Evol. Microbiol.* **73**, 005979 (2023). 10.1099/ijsem.0.005979 37486744

[30] Meier-Kolthoff, J. P. & Göker, M. TYGS is an automated high-throughput platform for state-of-the-art genome-based taxonomy. *Nat. Commun*. **10**, 2182 (2019). 10.1038/s41467-019-10210-3 PMC6522516 31097708

[31] Auch, A. F., Von Jan, M., Klenk, H.-P. & Göker, M. Digital DNA-DNA hybridization for microbial species delineation by means of genome-to-genome sequence comparison. *Stand. Genom. Sci.* **2**, 117–134 (2010). 10.4056/sigs.531120 PMC3035253 21304684

[32] Izawa, T. What is going on with the hormonal control of flowering in plants?. *Plant J.* **105**, 431–445 (2021). 33111430 10.1111/tpj.15036

[33] Noman Khan, M. & Nabi, G. Role of Auxin in vegetative growth, flowering, yield and fruit quality of Horticultural crops—a review. *Pure Appl. Biol.* **12**, 1234–1241 (2023).

[34] Taha, S. T., Kareem, A. & Saeed, A. J. M. Effect of spraying with putrescine and indole acetic acid on two strains of *Antirrhinum majus* L. *Euphrates. J. Agric. Sci*. **14**, 283–292 (2022).

[35] Topno, S., Meghana, L., Vidyullatha, M. & Topno, S. E. Vidyullatha and Topno. Effect of Naphthalene Acetic Acid & Indole Acetic Acid on Growth, Yield and Quality of Muskmelon (*Cucumis melo* L.). *IJPSS* **34**, 1460–1469 (2022).

[36] Mirheidari, F., Hatami, M. & Ghorbanpour, M. Effect of different concentrations of IAA, GA3 and chitosan nano-fiber on physio-morphological characteristics and metabolite contents in roselle (*Hibiscus sabdariffa* L.). *South Afr. J. Bot.* **145**, 323–333 (2022).

[37] Bernales, M. et al. Expression of two indole-3-acetic acid (IAA)-amido synthetase (GH3) genes during fruit development of raspberry (*Rubus idaeus* Heritage). *Sci. Hortic.* **246**, 168–175 (2019).

[38] Walker, C. H. & Bennett, T. Cytokinin and reproductive shoot architecture: bigger and better?. *Biochem. Soc. Trans.* **52**, 1885–1893 (2024). 39083016 10.1042/BST20231565 PMC11668285

[39] Ananev, A. A. et al. Whole genome sequencing of *Bacillus velezensis* AMR25, an effective antagonist strain against plant pathogens. *Microorganisms* **12**, 1533 (2024). 39203375 10.3390/microorganisms12081533 PMC11356610

[40] Azizul ISlAM, M. et al. ScienceDirect Putrescine, spermidine, and spermine play distinct roles in rice salt tolerance. *J. Integr. Agric.* **2020**, 643–655 (2020).

[41] González-Hernández, A. I. et al. Putrescine: a key metabolite involved in plant development, tolerance and resistance responses to stress. *Int. J. Mol. Sci.* **23**, 2971 (2022). 35328394 10.3390/ijms23062971 PMC8955586

[42] Krysenko, S. & Wohlleben, W. Polyamine and ethanolamine metabolism in bacteria as an important component of nitrogen assimilation for survival and pathogenicity. *Med. Sci.* **10**, 40 (2022). 10.3390/medsci10030040 PMC9397018 35997332

[43] Thongbhubate, K., Irie, K., Sakai, Y., Itoh, A. & Suzuki, H. Improvement of putrescine production through the arginine decarboxylase pathway in *Escherichia coli* K-12. *AMB Express* **11**, 1–13 (2021). 34910273 10.1186/s13568-021-01330-5 PMC8674398

[44] Dai, H. et al. Putrescine treatment delayed the softening of postharvest blueberry fruit by inhibiting the expression of cell wall metabolism key gene VcPG1. *Plants* **11**, 1356 (2022). 35631781 10.3390/plants11101356 PMC9143846

[45] Zhang, Y., Liu, B., Kong, F. & Chen, L. Nutrient-mediated modulation of flowering time. *Front. Plant Sci.* **14**, 1101611 (2023). 36743493 10.3389/fpls.2023.1101611 PMC9894683

[46] Jiang, L., Strobbe, S., Van Der Straeten, D., Zhang, C. & Der Straeten, V. D. Regulation of plant vitamin metabolism: backbone of biofortification for the alleviation of hidden hunger. *Mol. Plant* **14**, 40–60 (2021). 10.1016/j.molp.2020.11.019 33271336

[47] Averianova, L. A., Balabanova, L. A., Son, O. M., Podvolotskaya, A. B. & Tekutyeva, L. A. Production of vitamin B2 (Riboflavin) by microorganisms: an overview. *Front. Bioeng. Biotechnol.* **8**, 570828 (2020). 33304888 10.3389/fbioe.2020.570828 PMC7693651

[48] D’aimmo, M. R. et al. Folate-producing bifidobacteria: metabolism, genetics, and relevance. *Microbiome Res. Rep.* **12**, 3 (2024). 10.20517/mrr.2023.59 PMC10917623 38455078

[49] López-Sámano, M. et al. A novel way to synthesize pantothenate in bacteria involves β-alanine synthase present in uracil degradation pathway. *Microbiol. Open* **9**, e1006 (2020). 10.1002/mbo3.1006 PMC7142369 32112625

[50] Xu, S. et al. Enhanced cobalamin biosynthesis in *Ensifer adhaerens* by regulation of key genes with gradient promoters. *Synth. Syst. Biotechnol.* **7**, 941–948 (2022). 10.1016/j.synbio.2022.04.012 PMC9157374 35664931

[51] Yahia, E. M. *Fruit and Vegetable Phytochemicals: Chemistry and Human Health* 2nd edn, Vol. 1, 1–1405 (John Wiley & Sons, 2017).

[52] Pandey, S. & Tyagi, D. N. Changes in chlorophyll content and photosynthetic rate of four cultivars of mango during reproductive phase. *Biol. Plant.* **42**, 457–461 (1999).

[53] Schaffer, B., Barden, J. A. & Williams, J. M. Net photosynthesis, dark respiration, stomatal conductance, specific leaf weight, and chlorophyll content of strawberry plants as influenced by fruiting. *J. Am. Soc. Hortic. Sci.* **111**, 82–86 (1986).

[54] Vemmos, S. N. Net photosynthesis, stomatal conductance, chlorophyll content and specific leaf weight of pistachio trees (cv. Aegenes) as influenced by fruiting. *J. Hortic. Sci.* **69**, 775–782 (1994).

[55] Cosme, F. et al. Red fruits composition and their health benefits—a review. *Foods* **11**, 644 (2022). 10.3390/foods11050644 PMC8909293 35267278

[56] Larrosa, M. et al. Anti-inflammatory properties of a pomegranate extract and its metabolite urolithin-A in a colitis rat model and the effect of colon inflammation on phenolic metabolism. *J. Nutr. Biochem.* **21**, 717–725 (2010). 19616930 10.1016/j.jnutbio.2009.04.012

[57] Vuolo, M. M., Lima, V. S. & Maróstica Junior, M. R. Phenolic compounds: structure, classification, and antioxidant power. in *Bioactive Compounds Health Benefits and Potential Applications,* (ed. Segura-Campos, M.), pp. 33–50 (Woodhead Publishing, 2019).

[58] Gupta, R., Saikia, S. K. & Pandey, R. Bioconsortia augments antioxidant and yield in *Matricaria recutita* L. against *Meloidogyne incognita* (Kofoid and White) Chitwood Infestation. *Proc. Natl. Acad. Sci. India Sect. B Biol. Sci.* **87**, 335–342 (2017).

[59] Flores-Félix, J. D. et al. *Rhizobium* as plant probiotic for strawberry production under microcosm conditions. *Symbiosis* **67**, 25–32 (2015).

[60] Liu, L. et al. The review of anti-aging mechanism of polyphenols on *Caenorhabditis elegans*. *Front. Bioeng. Biotechnol.* **9**, 635768 (2021). 34327192 10.3389/fbioe.2021.635768 PMC8314386

[61] Rahman, S., Mathew, S., Nair, P., Ramadan, W. S. & Vazhappilly, C. G. Health benefits of cyanidin-3-glucoside as a potent modulator of Nrf2-mediated oxidative stress. *Inflammopharmacology* **29**, 907–923 (2021). 33740221 10.1007/s10787-021-00799-7

[62] Periferakis, A. et al. Kaempferol: antimicrobial properties, sources, clinical, and traditional applications. *Int. J. Mol. Sci.* **23**, 15054 (2022). 36499380 10.3390/ijms232315054 PMC9740324

[63] Maldonado, E., Morales-Pison, S., Urbina, F. & Solari, A. Aging hallmarks and the role of oxidative stress. *Antioxidants* **12**, 651 (2023). 36978899 10.3390/antiox12030651 PMC10044767

[64] Miranda-Vizuete, A. & Veal, E. A. *Caenorhabditis elegans* as a model for understanding ROS function in physiology and disease. *Redox Biol.* **11**, 708 (2017). 28193593 10.1016/j.redox.2016.12.020 PMC5304259

[65] Tomás-Barberán, F. A. & Clifford, M. N. Review Dietary hydroxybenzoic acid derivatives-nature, occurrence and dietary burden. *J. Sci. Food Agric.* **80.7**, 1024–1032 (2000).

[66] Ayuda-Durán, B. et al. Epicatechin modulates stress-resistance in *C. elegans* via insulin/IGF-1 signaling pathway. *PLoS ONE* **14**, e0199483 (2019). 30689636 10.1371/journal.pone.0199483 PMC6349306

[67] Moraes, D. P. et al. Characterization of a new blackberry cultivar BRS Xingu: chemical composition, phenolic compounds, and antioxidant capacity in vitro and in vivo. *Food Chem.* **322**, 126783 (2020). 32305870 10.1016/j.foodchem.2020.126783

[68] Moreno-Medina, B. L., Casierra-Posada, F. & Medina-Vargas, O. J. Phenolic profile and antioxidant capacity of blackberry fruits (*Rubus* spp) grown in Colombia. *Erwerbs-Obstbau* **65**, 1047–1056 (2023).

[69] Zhang, N., Jiao, S. & Jing, P. Red cabbage rather than green cabbage increases stress resistance and extends the lifespan of *Caenorhabditis elegans*. *Antioxidants* **10**, 930 (2021). 10.3390/antiox10060930 PMC8228718 34201067

[70] Li, R. et al. Food & Function REVIEW Small berries as health-promoting ingredients: a review on anti-aging effects and mechanisms in *Caenorhabditis elegans. Food Funct.* **13**, 478–500 (2022). 10.1039/d1fo02184b 34927654

[71] Tian, R., Seim, I., Ren, W., Xu, S. & Yang, G. Contraction of the ROS scavenging enzyme glutathione S-transferase gene family in cetaceans. *G3 Genes Genomes Genet.* **9**, 2303 (2019). 10.1534/g3.119.400224 PMC6643896 31092607

[72] Ye, Y., Gu, Q. & Sun, X. Potential of *Caenorhabditis elegans* as an antiaging evaluation model for dietary phytochemicals: a review. *Compr. Rev. Food Sci. Food Saf.* **19**, 3084–3105 (2020). 33337057 10.1111/1541-4337.12654

[73] Gutierrez-Zetina, S. M., González-Manzano, S., Ayuda-Durán, B., Santos-Buelga, C. & González-Paramás, A. M. Caffeic and dihydrocaffeic acids promote longevity and increase stress resistance in *Caenorhabditis elegans* by modulating expression of stress-related genes. *Molecules* **26**, 1517 (2021). 33802064 10.3390/molecules26061517 PMC8001149

[74] Song, B., Zheng, B., Li, T. & Liu, R. H. Raspberry extract promoted longevity and stress tolerance via the insulin/IGF signaling pathway and DAF-16 in *Caenorhabditis elegans*. *Food Funct.* **11**, 3598–3609 (2020). 32285078 10.1039/c9fo02845e

[75] Tambara, A. L. et al. Purple pitanga fruit (*Eugenia uniflora* L.) protects against oxidative stress and increase the lifespan in *Caenorhabditis elegans* via the DAF-16/FOXO pathway. *Food Chem. Toxicol.* **120**, 639–650 (2018). 30077708 10.1016/j.fct.2018.07.057

[76] Rivas, R. et al. A new species of *Devosia* that forms a unique nitrogen-fixing root-nodule symbiosis with the aquatic legume *Neptunia natans* (L.f.) Druce. *Appl. Environ. Microbiol.* **68**, 5217–5222 (2002). 12406707 10.1128/AEM.68.11.5217-5222.2002 PMC129907

[77] Gonçalves, A. C. et al. Insight into the taxonomic and functional diversity of bacterial communities inhabiting blueberries in Portugal. *Microorganisms* **10**, 2193 (2022). 36363783 10.3390/microorganisms10112193 PMC9695653

[78] Pikovskaya, R. I. Mobilization of phosphorus in soil in connection with the vital activity of some microbial species—ScienceOpen. *Mikrobiologiya* **17**, 362–370 (1948).

[79] Nautiyal, C. S. An efficient microbiological growth medium for screening phosphate solubilizing microorganisms. *FEMS Microbiol. Lett.* **170**, 265–270 (1999). 9919677 10.1111/j.1574-6968.1999.tb13383.x

[80] Aleksandrov, V. G., Blagodyr, R. N. & Illev, I. P. Liberation of phosphoric acid from apatite by silicate bacteria. *Mikrobiol. Zh.* **29**, 111–114 (1967). 4308691

[81] Alexander, D. B. & Zuberer, D. A. Use of chrome azurol S reagents to evaluate siderophore production by rhizosphere bacteria. *Biol. Fertil. Soils* **12**, 39–45 (1991).

[82] O’Hara, G. W., Goss, T. J., Dilworth, M. J. & Glenn, A. R. Maintenance of intracellular pH and acid tolerance in *Rhizobium meliloti*. *Appl. Environ. Microbiol.* **55**, 1870–1876 (1989). 16347984 10.1128/aem.55.8.1870-1876.1989 PMC202972

[83] Jiménez-Gómez, A. et al. Probiotic activities of *Rhizobium laguerreae* on growth and quality of spinach. *Sci. Rep.* **8**, 295 (2018). 29321563 10.1038/s41598-017-18632-z PMC5762915

[84] Zerbino, D. R. & Birney, E. Velvet: algorithms for de novo short read assembly using de Bruijn graphs. *Genome Res.* **18**, 821–829 (2008). 18349386 10.1101/gr.074492.107 PMC2336801

[85] Overbeek, R. et al. The SEED and the rapid annotation of microbial genomes using subsystems technology (RAST). *Nucleic Acids Res.* **42**, D206 (2014). 24293654 10.1093/nar/gkt1226 PMC3965101

[86] Patz, S. et al. PLaBAse: a comprehensive web resource for analyzing the plant growth-promoting potential of plant-associated bacteria. *bioRxiv*. 10.1101/2021.12.13.472471 (2021).

[87] Alcalde-Eon, C. et al. Adding oenological tannin vs. overripe grapes: Effect on the phenolic composition of red wines. *J. Food Compos. Anal.* **34**, 99–113 (2014).

[88] Alcalde-Eon, C., García-Estévez, I., Puente, V., Rivas-Gonzalo, J. C. & Escribano-Bailón, M. T. Color stabilization of red wines. A chemical and colloidal approach. *J. Agric. Food Chem.* **62**, 6984–6994 (2014). 24593183 10.1021/jf4055825

