# Supplementary material for: Rhizobium biostimulation of blackberry modulates survival pathways in Caenorhabditis elegans across biological kingdoms
Source: NPJ Sci Food. 2025 Jul 29;9:160. doi: 10.1038/s41538-025-00525-5 (PMC12307951; doi:10.1038/s41538-025-00525-5)
Supplement: Supplementary file 1 — Supplementary Material. [file 41538_2025_525_MOESM1_ESM.docx]

**SUPPLEMENTARY MATERIAL**

**Supplementary Table 1.** Composition of the NGM medium. Components marked with an asterisk are sterilized by filtration and added after autoclaving the medium.

| NGM (Nematode Growth Medium) | |
| --- | --- |
| NaCl | 1.2 g |
| Peptone | 1 g |
| CaCl_2_ 1 M pH 6,0 | 400 µL |
| Cholesterol (5 mg/mL ethanol) | 400 µL |
| MgSO_4_ 1 M * | 400 µL |
| Buffer phosphate 1 M pH 6,0 | 400 µL |
| Sodium ampicilin (50 µg/mL) | 400 µL |
| Nistatin (1 g/100 mL in ethanol: ammonium acetate 1:1) | 700 µL |
| Agar | 6,8 g |
| H_2_O | 390 mL |

**Supplementary Table 2.** Genome characteristics of *Rhizobium* sp. CRRU65 genome.

| Characteristics | *Rhizobium* sp*.* CRRU65 |
| --- | --- |
| Size | 7,539,570 |
| GC Content (%) | 60.7 |
| N50 | 280,285 |
| L50 | 8 |
| Nº *contigs* (with PEGs) | 87 |
| Nº subsystems | 366 |
| Nº coding sequences | 7,743 |
| Nº ARNs | 49 |
| Completeness (%) | 99.8 |
| Contamination (%) | 0.16 |

**Supplementary Table 3**. Compounds, retention times (Rt), maximum absorption spectra (λ_max_), mass spectra results and quatification (µg/g d.w.) of (poly)phenolics identified in blackberry fruits.

| Peak | λmax (nm) | Rt (min) | Molecular ion  [M -H] [M +H]* (m/z) | Fragments MSn (m/z) | Compound | Control | CRRU65 |
| --- | --- | --- | --- | --- | --- | --- | --- |
| 1 | 280 | 6,75 | 198 | 163 | Unknown | 0.398 ± 0.076 a | 0.521 ± 0.013 a |
| 2 | 324 | 10,78 | 353 | 169 | 3-Caffeoylquinic acid | 0.182 ± 0.020 a | 0.213 ± 0.028 a |
| 3 | 280 | 15,92 | 198 | 163 | Unknown | 2.548 ± 0.096 a | 3.196 ± 0.174 a |
| 4 | 274 | 17,61 | 937 | 465/301 | Trigalloyl HHDP glucoside | 20.516 ± 0.494 b | 24.120 ± 2.420 a |
| 5 | 360 | 18,53 | 331 | 169 | Glucogallin | 10.043 ± 1.215 a | 4.628 ± 0.433 b |
| 6 | 520 | 19,69 | 585 | 465/301 | Derivative of Cyanidin-3-o-pentoside | 2.433 ± 0.168 a | 3.138 ± 0.275 a |
| 7 | 281 | 21,21 | 577 | 425/407/289 | Dimer of procyanidin type B | 3.014 ± 0.182 a | 2.910 ± 0.384 a |
| 8 | 343 | 22,01 | 346 | 327/287 | Kaempferol acetylhexoside | 0.441 ± 0.0772 b | 0.766 ± 0.0324 a |
| 9 | 278 | 24,84 | 289 | 289 | Epicatequin | 11.122 ± 0.771a | 9.000 ± 0.845 a |
| 10 | 288 | 26,04 | 449 | 287 | Eridictyol hexoside | 0.398 ± 0.076 a | 0.521 ± 0.130 a |
| 11 | 356 | 26,29 | 477 | 301 | Quercertin-3-O-glucuronide | 0.309 ± 0.118 a | 0.256 ± 0.049 a |
| 12 | 516 | 26,68 | 449 | 287 | Cyanidin-3-o-glucoside | 41.337 ± 3.684 b | 46.115 ± 2.422 a |
| 13 | 356 | 28,9 | 419 | 287 | Cyanidin-3-o-pentoside | 4.127 ± 0.460 a | 3.659 ± 0.180 a |
| 14 | 285 | 32,7 | 934 | 631/469/301 | Sanguiin H6 | 180.960 ± 7.071 b | 215.279 ± 17.677 a |
| 15 | 353 | 32,82 | 433 | 287 | Cyanidin-3-o-pentoside | 0.218 ± 0.043 a | 0.156 ± 0.027a |
| 16 | 285 | 35,05 | 934 | 631/469/301 | Isomer of Sanguiin H6 | 383.954 ± 16.112 b | 441.678 ± 20.469 a |
| 17 | 520 | 36,91 | 419 | 271 | Pelargonidin-3-O- glucoside | 1.755 ± 0.161 a | 1.839 ± 0.337 a |
| 18 | 360 | 37,96 | 433 | 301 | Pentoside of ellagic acid | 5.484 ± 0.612 a | 6.047 ± 0.308 a |
| 19 | 518 | 38,5 | 535 | 287 | Cyanidin-3-o-malonilglucoside | 0.579 ± 0.011 a | 0.462 ± 0.036 a |
| 20 | 350 | 39,4 | 593 | 287 | Kaempferol rutinoside | 0.256 ± 0.032 a | 0.300 ± 0.032 a |
| 21 | 364 | 39,7 | 609 | 301 | Quercetin-3-O-rutinoside | 0.335 ± 0.012 a | 0.275 ± 0.030 a |
| 22 | 360 | 40,07 | 301 | 301 | Ellagic acid | 5.186 ± 0.452 a | 6.511 ± 0.338 a |
| 23 | 356 | 40,3 | 463 | 301 | Quercetin hexoside | 0.791 ± 0.069 a | 0.765 ± 0.106 a |
| 24 | 356 | 41,07 | 463 | 191/161 | Quercetin hexoside | 0.478 ± 0.044 a | 0.442 ± 0.038 a |
| 25 | 356 | 43,41 | 505 | 301 | Quercetin acetylhexoside | 0.497 ± 0.046 a | 0.474 ± 0.050 a |
